# ORTHOPAEDIC MANUAL PHYSICAL THERAPY FOR POST-STROKE SHOULDER PAIN: TWO CASE REPORTS INCLUDING CLINICAL ASSESSMENTS AND PARTICIPANTS’ EXPERIENCES

**DOI:** 10.2340/jrm-cc.v8.43201

**Published:** 2025-06-03

**Authors:** Ingrid LINDGREN, Arne G. LINDGREN, Håkan CARLSSON, Tobias LUNDGREN, Christina BROGÅRDH

**Affiliations:** 1 Department of Health Sciences, Lund University, Lund, Sweden; 2 Department of Neurology, Rehabilitation Medicine, Memory Disorders and Geriatrics, Skåne University Hospital, Lund, Sweden; 3 Department of Clinical Sciences Lund, Neurology, Lund University, Lund, Sweden; 4 Palmer Friskvård, Malmö, Sweden

**Keywords:** stroke, post-stroke shoulder pain, orthopaedic manual physical therapy, case report, physiotherapy

## Abstract

**Objective:**

To descriptively evaluate Orthopaedic Manual Physical Therapy – a novel intervention for post-stroke shoulder pain – by use of clinical assessments and the participants’ experiences.

**Design:**

Two case reports.

**Methods:**

Two individuals with mild to moderate upper extremity impairments and persistent post-stroke shoulder pain, underwent Orthopaedic Manual Physical Therapy for 12 weeks. The intervention comprised a thorough clinical examination, joint mobilization, stretching, and exercises targeting the affected structures and incorrect movement patterns. Participants were clinically assessed pre- and post-intervention and followed up 4–5 months later. They also answered interviews about their experiences of the intervention and perceived effects.

**Results:**

After Orthopaedic Manual Physical Therapy, both participants showed decreased pain intensity during movements and increased range of motion. One of the participants also experienced decreased resistance to passive movements, improved motor function, grip strength, and upper extremity daily activities after the intervention and at follow-up. Interviews revealed that the participants tolerated the therapy well and were satisfied with the intervention and long-lasting results.

**Conclusion:**

Orthopaedic Manual Physical Therapy may be a useful method to reduce post-stroke shoulder pain in persons with mild to moderate upper extremity paresis after stroke. To confirm the results, further studies are warranted.

## LAY ABSTRACT

Although shoulder pain could be a disabling condition in persons with arm paresis after stroke, the effectiveness of existing interventions is limited. Orthopaedic Manual Physical Therapy is a promising intervention for persons with orthopedic shoulder related injuries. In Orthopaedic Manual Physical Therapy, a thorough examination leads to a clinical diagnosis. The intervention is individualized, specifically targeting the person’s affected structures and incorrect movement patterns. In this study, we evaluated 12 weeks of Orthopaedic Manual Physical Therapy in 2 persons with shoulder pain after stroke. After the intervention, we observed decreased pain intensity and increased arm mobility in both participants. One of the participants also showed improvements in grip strength and upper extremity daily activities. The participants expressed that they tolerated the intervention well and were satisfied with the intervention and long-lasting results. Thus, Orthopaedic Manual Physical Therapy might be a useful intervention for persons with post-stroke shoulder pain, but larger studies are warranted.

After stroke, various impairments in the upper extremity (UE) are common, such as reduced sensorimotor function (1), decreased range of motion (ROM) (2), spasticity (3) and post-stroke shoulder pain (PSSP) (4, 5). PSSP is reported by 22–47% of persons with stroke (6), and is more common among those with severe and persistent UE impairments (4, 7). The PSSP most often occurs within the first weeks or months after stroke (4, 7). For some, the pain decreases over time, but about 70% of those who develop PSSP within a few months after stroke onset, still have pain after 1 year (4, 5, 8). Activity limitations are reported to be more common in persons with PSSP than in persons without PSSP (9–11). Also, an association between PSSP and decreased participation (12) as well as quality of life (13, 14) has been reported.

The underlying causes of PSSP are considered to be multifactorial (15, 16). Factors related to the pain include impaired UE motor function (4, 5, 8), decreased passive ROM (2, 5, 17, 18), somatosensory impairments (19), and spasticity (20, 21). Possible conditions that may contribute to PSSP comprise soft tissue lesions (impingement), rotator cuff or bicipital tendinopathy, and adhesive capsulitis (15). Moreover, instability and muscle weakness around the shoulder have also been associated with PSSP (4, 22).

To reduce PSSP, a variety of interventions, both pharmacological and non-pharmacological, are suggested in the literature (23). Examples of pharmacological therapies are oral analgesic medication, intra-articular injections (24), and botulinum toxin injections (25, 26). Non-pharmacological therapies include acupuncture, strapping (25), orthosis (25, 27), electrical stimulation (25, 28) and positioning of the arm (29). In clinical practice, a combination of interventions is often used. However, evidence of the effectiveness of the interventions is limited. Thus, there is a great need to develop more efficient rehabilitation interventions to reduce PSSP.

Orthopaedic Manual Physical Therapy (OMPT) is a specialized area of physiotherapy for management of neuro-musculoskeletal conditions. OMPT is based on clinical reasoning, using highly specific treatment approaches including manual techniques and therapeutic exercises. It encompasses and is driven by the available scientific and clinical evidence and the biopsychosocial framework of each individual patient (30). OMPT is used for treatments of all types of musculoskeletal and/or peripheral neurological conditions, but is not routinely used in the rehabilitation of people with central neurological conditions.

However, positive experiences of OMPT to reduce PSSP have been found in clinical settings, but so far, no study has evaluated its effect. In this study, we report 2 cases with PSSP and UE impairments who underwent OMPT. Our aim was to descriptively evaluate the OMPT by use of clinical assessments and the participants’ experiences.

## MATERIAL AND METHOD

The current study is explorative in nature. Clinical assessments were performed pre- and post-intervention, and at follow-up 4 to 5 months later. At follow-up, an interview with the participants was also performed (Table I). The 2 participants were recruited from Skåne University Hospital, Sweden. A written document describing the study was sent to them; thereafter, written informed consent was obtained. The study was approved by the Swedish Ethical Review Authority (2016/179, 2018/345) and the principles of the Declaration of Helsinki were followed.

**Table I:** Timeline for inclusion, assessments and intervention

Stroke onset >----->	Inclusion >-----> Pre-intervention assessments	Clinical >------> diagnosis	Intervention >------>	Post-intervention assessments >------>	Follow-up assessments
	6–9 months after stroke onset	About a week after inclusion	Two times a week during 12 weeks	About a week after the intervention	4–5 months after the intervention
	Clinical assessments performed by a physiotherapist not involved in the intervention	Clinical assessments leading to a clinical diagnosis of which the individualized, tailored treatment and exercise plan was based, performed by the treating OMPT physiotherapist	Individualized intervention, performed by the treating OMPT physiotherapist Home exercises	Same clinical assessments as at inclusion, performed by a physiotherapist not involved in the intervention	Same clinical assessments as at inclusion performed by a physiotherapist not involved in the intervention Interview

OMPT: Orthopaedic Manual Physical Therapy.

### Assessments and interviews

The clinical assessments took place in an outpatient setting, and were performed by a skilled physiotherapist with knowledge and experience of stroke rehabilitation and OMPT (who was not involved in the intervention). The clinical assessments included registration of:

Shoulder pain characteristics (pain location, pain frequency, pain character) (31) and shoulder pain intensity during rest/movements, (scored between 0 and 100 mm on the Visual Analogue Scale for Pain (VAS-P) (in Swedish) (32); active and passive ROM in flexion and abduction of the shoulder assessed by a hand held digital goniometer (33); subacromial impingement assessed by the Hawkins-Kennedy test (34); resistance to passive movements in the elbow, measured by the Modified Ashworth Scale, MAS (35, 36); motor function in the arm and hand, measured by Modified Motor Assessment Scale (M-MAS, Swedish version) (37, 38); grip strength, assessed with Jamar dynamometer (39); light touch and joint position in the arm and hand, assessed with the sensory part of the Fugl-Meyer UE test (40, 41) and ability to use the arm in daily activities, assessed by the Motor Activity Log (MAL) (42–44).

Four months (Particpant 2) and 5 months (Particpant 1) post-intervention, the participants were interviewed by telephone by the first author (IL). The interviews were based on a semi-structured guide and focused on how the participants experienced the intervention and its effects. Examples of questions were: ‘Could you describe how you perceived the training?’ and ‘Did you perceive any effect of the training? In what way?’ Supplementary questions were added when needed. The interviews lasted around 20 min each. They were digitally recorded and transcribed verbatim by the first author (IL). The interviews were analysed with manifest content analysis according to Graneheim and Lundman (45), by the first author (IL) in collaboration with HC and CB. The transcribed interviews were first read through several times, to get an overview of the whole. Thereafter, all content that responded to the perceptions of the OMPT intervention was identified as meaning units. The meaning units were coded and sorted into subcategories and categories. To add transparency and trustworthiness to the findings, quotations were added.

### Intervention

Pre-intervention, the treating physiotherapist, an OMPT specialist with experience of stroke rehabilitation, made a thorough clinical examination of the participants (Table I). This included inspection of different positions of the arm/shoulder, examinations of ROM and quality of movement during active and passive movements, tests for shoulder instability, muscle strength, nerves, and joints as well as tests of surrounding structures. The examination led to a clinical diagnosis of which the individualized, tailored treatment and exercise plan was based. The intervention was individualized to each participant’s specific problems (see below) but could include mobilization of shortened structures and joints around the shoulder, neuromuscular activation and movement control of stabilizing muscles, and of endurance training. The intervention was performed with personal supervision by the treating physiotherapist 2 times a week, 30–45 min sessions, for about 12 weeks. The number of repetitions for each exercise ranged between 20 and 30. After the 12-week OMPT period, no additional specific training recommendations were given to the participants.

### Particpant 1

*Description.* Participant 1 was a 48-year-old woman who had a stroke about 9 months earlier. She lived by herself, had some walking difficulties but could walk independently. She had a moderate paresis in her right arm with increased muscle tone. The PSSP developed within 2 months from the stroke onset. Before OMPT she had undergone multidisciplinary inpatient rehabilitation, that was not specifically focused on PSSP. The participant described that she could not do much with her arm; it was stiff and very painful. She did not use analgesic medication. The clinical diagnoses set by the treating OMPT physiotherapist were bicipital tendinopathy and subacromial impingement syndrome.

*Individualized intervention.* The OMPT for Particpant 1 included transverse friction massage (46, 47), stretching (47), capsular mobilization and thoracic mobilization (46, 47), aiming at decreasing stiffness in the levator scapulae, rhomboid, infraspinatus and teres minor muscles. Also, glenohumeral joint mobilization and thoracic mobilization of costotransversal joints and ribs were performed to allow scapulae to rotate upwards during UE movements. Moreover, guided and partially weight-bearing exercises in a cable pulley machine were performed. The cable pulley training aimed to prevent synergetic position of protraction and forward tilt of the scapula, elbow flexion and forearm pronation.

### Participant 2

*Description.* Participant 2 was a 66-year-old man who had a stroke about 6 months earlier. He lived by himself had some walking difficulties but could walk independently. He had a mild paresis in his right arm. The PSSP developed a few weeks after the stroke. Before OMPT, he had undergone multidisciplinary outpatient rehabilitation, that was not specifically focused on PSSP. He did not use any analgesic medication. The clinical diagnoses set by the treating OMPT physiotherapist were decreased glenohumeral mobility and compensatory overuse of the scapula retractors and the shoulder external rotators, which caused friction tendinosis of the biceps longus tendon.

*Individualized intervention.* The OMPT for Particpant 2 included muscular soft tissue treatment (46, 47) to decrease muscle tone. Also, stretching (47) was used in different degrees of abduction to release tension in pectoral muscles, latissimus dorsi, anterior deltoid and bicep brachii. In supine lying, with various degrees of flexion, soft tissue treatment and stretching of external rotators, rhomboids, levator scapulae and posterior deltoid muscles were performed. Treatment of the same muscles was repeated in the supine position with pain free hand in the neck position. The subject also performed home exercises, comprising stretching for increased mobility, and small active exercises for correcting movement patterns rather than utilising the full arm ROM.

## Results

The results from the clinical assessments for the 2 participants pre- and post-intervention and at 4–5 months follow-up, as well as findings from the interviews, are presented below and shown in Tables II and III.

**Table II:** Shoulder pain and functioning of upper extremity for Participant 1 and Participant 2

Clinical assessments	Participant 1			Participant 2		

	Pre-intervention	Post-intervention	Follow-up	Pre-intervention	Post-intervention	Follow-up
Pain at rest/movement^a^, mm	15/72	0/0	20/40	0/78	0/12	0/0
Shoulder active/passive flexion^b^, degrees	47/83	130/147	140/145	162/162	180/180	180/180
Shoulder active/passive abduction^b^, degrees	39/61	93/90	150/150	168/168	180/180	180/180
Subacromial impingement^c^, yes/no	Yes	No	No	Yes	No	No
Resistance to passive movement in the elbow^d^, grade	2	1	1	0	0	0
Upper extremity motor function^e^, points	0	3	7	15	15	15
Hand grip strength^f^, kg	2	8	14	48	50	49
Ability to use the arm in daily activities^g^, points						
Amount of arm use	29	32	42	150	149	149
Quality of movement	18	33	37	149	149	148

a VAS-P: Visual Analogue Scale – Pain, (0–100 mm);

b degrees assessed with a digital goniometer;

c Hawkins-Kennedy test, yes/no;

d modified Ashworth Scale;

e Modified Motor Assessment Scale, M-MAS UAS, UE (0–15 points);

f Jamar, kilogrammes;

g MAL: Motor Activity Log (0–150 points).

Follow-up was performed 4–5 months after the completed Orthopaedic Manual Physical Therapy period.

**Table III:** The results from the semi-structured interview at follow-up, analysed with manifest qualitative content analysis, presented in categories and illustrated by quotations in Italic (translated from Swedish to English)

**Experience of the intervention** The participants perceived that the instructions to movements were easy to follow, and that the intervention was extremely focused on the actual shoulder problem.	
Participant 1 *I noticed that when I performed these movements [for the shoulder; upwards, sideways, with weights]… it didn’t take long for the brain to get the hang of these movements.*	Participant 2 *…the person [the physiotherapist] pulled and bent a little bit in the shoulder to soften it, to make the muscle relax. And it was very, very efficient… He [the physiotherapist] put his fingers down and pushed for 10–15 seconds, massaging with his fingertips under my scapular muscles … [And] he’d say: – Would you look at that, things are starting to happen! And I’ll be damned, I started to feel it wearing off… when I started to be able to move my arm, we sped it up a little [so that] I’d be able to move a bit more.*
**Adverse effects after the training sessions** Participant 1 expressed that she never had pain after the training sessions. But, she experienced being very tired as she suffered from fatigue. Participant 2 described some pain during the training sessions, but he learned to relax and rely on the physiotherapist as the pain decreased gradually.	
Participant 1 *No… well like a few times, when he pushed, it could hurt, but not worse than, no, no… He said that it might hurt for a few days, but it never did.* *[The training] was okay… I noticed that I got tired, yeah, this fatigue, I noticed that. Yeah, I slept for two hours [after the training session].*	Participant 2 *It’s a nice experience going to a place like that, here I am having problems with a radiating pain in my arm and an unpleasant feeling when I move my arm in this position. And when I leave, it’s much, much better. So, it’s a positive thing that actually gives you energy instead.*
**Intervention effects** The participants described that after the OMPT period, the pain was absent. Participant 1 also perceived that the muscle tone had decreased, and that the arm was stronger. Participant 2 expressed that the affected arm had regained even better range of motion than the unaffected arm.	
Participant 1 *It [the intervention] helped me a lot, I don’t feel any pain anymore… and I can swing [my arm around]… I can lift my arm straight up [and] I always open the cabinets with this [affected] arm… up there, I always open those with this [affected arm]…Before, when I vaccuumed, I would only use one arm. Now I use the other one as well… it’s gotten a bit stronger too.*	Participant 2 *I’ll be damned, my range of motion is better on my right side* [shoulder] *which was my affected one, than on my left side… [and] it’s a big difference [in the ability to use the arm and hand in daily activities], because now I can move it… so it’s gotten better for sure, there’s no denying that, it’s really good now.* *I didn’t really think it [the training] would have the impact it actually had. I’m very thankful I got the opportunity to be a part of this project. And to get that help, because I don’t think it would be possible to train it up with physical training… with weights and such… there’s no comparing it, the difference in how much better I’ve become after getting help with all of this…*

OMPT: Orthopaedic Manual Physical Therapy.

### Results from clinical assessments Participant 1

Pre-intervention, Particpant 1 had constant PSSP (i.e. pain both day and night). The pain was described as “like a knife,” and she had also tingling and numbness in the fingers. Light touch was intact in the arm, but diminished in the hand and fingers. Joint position was registered as intact in the wrist and as 1 (3/4 attempts correct) in the thumb. The pain was rated to VAS-P 15/72 mm during rest/movements. Active shoulder flexion and abduction were below 50 degrees, and passive flexion and abduction were below 90 degrees. Subacromial impingement occurred during the Hawkins-Kennedy test. Resistance to passive movements was registered as grade 2 on the Modified Ashworth scale. Also, reduced strength and ability to use the arm and hand in daily activities were registered. Post-intervention, the pain had decreased and was rated 0 mm in VAS-P both during movements and at rest. The ROM in shoulder flexion and abduction had increased both actively and passively. Joint position was registered as intact. No impingement was revealed in the Hawkins-Kennedy test. Resistance to passive movements had decreased to grade 1 according to the Modified Ashworth scale. Also, motor function in the UE, the ability to use the arm in daily activities and grip strength had increased. At follow-up, some pain during movements was present. Motor function in the UE, the ability to use the arm in daily activities, and grip strength had further increased since the end of intervention (Table II).

### Results from clinical assessments Participant 2

Pre-intervention, Particpant 2 often had shoulder pain at daytime and avoided lying on the paretic side because of the pain. The PSSP was described as burning, with pain radiating to the arm. Sensory function was intact. The pain was rated to VAS-P 78 mm during movements, and subacromial impingement was present at the Hawkins-Kennedy test. Active and passive ROM in flexion and abduction was reduced about 15–20 degrees. Post-intervention, the pain had decreased during movements to VAS-P 12 mm, and ROM in flexion and abduction had increased. Impingement was no longer present at the Hawkins-Kennedy test. Motor function and grip strength were still good, as well as the ability to use the hand in daily activities. At follow-up, the pain had completely disappeared. The other outcomes were similar in comparison with the assessments performed after the intervention (Table II).

### Interviews at follow-up

The semi-structured interviews verified the results from the clinical assessments. Both participants were satisfied with the OMPT and perceived that the intervention was extremely focused on their actual shoulder problem, with negligible adverse effects after the training sessions. They experienced a long-lasting effect of the OMPT with decreased pain and increased ROM in the shoulder, as well as improved ability to use the UE in daily life (Table III).

## DISCUSSION

The aim of this study was to descriptively evaluate the effect of 12 weeks of OMPT in 2 persons with PSSP by use of clinical assessments and the participants’ experiences. Post-intervention, pain intensity was absent or greatly reduced in both participants. Improvements were seen in ROM, and none had any signs of impingement. For Particpant 1, resistance to passive movements was also decreased, and improvements were seen in motor function, grip strength and UE daily activities. The interviews revealed that the participants tolerated the OMPT well. They were satisfied with the intervention and the long-lasting results in terms of decreased pain and improved functioning of the arm.

The shoulder girdle is complex from an anatomical view, and changed position of the shoulder girdle due to muscle weakness after stroke might play an important role for the development and maintenance of PSSP. In case of spasticity, the increase in muscle tone combined with muscle weakness can easily lead to shortened structures and impingement. Also, in those with PSSP and mild UE motor impairments, delayed activitation, and inactivity of specific shoulder muscles, such as the infraspinatus, trapezius, and serratus anterior muscles have been reported during arm movements (48, 49). As the shoulder girdle is movable rather than stable, even small changes in the structures’ positions and movements might cause pain.

The positive results from the OMPT may have several explanations. The thorough examination performed pre-intervention by the treating physiotherapist, led to a clinical diagnosis, which was a prerequisite for the individualized, tailored treatment and exercise plan. The participants in this study were highly motivated, and could follow instructions. They perceived that the OMPT focused on their PSSP problems, confirming that the examination had identified the affected structures and impairments. In neurological rehabilitation of PSSP, often more general assessments are performed. In a survey from the UK (50), physical and occupational therapists described how they assessed, diagnosed, and managed PSSP. The most frequently reported assessments were related to pain, glenohumeral subluxation, ROM, spasticity, and strength. Similar results were found in a review (51). Such assessments might be blunt and not detailed enough to get an understanding of which structures cause the PSSP in the individual patient.

The clinical diagnoses set by the treating physiotherapist were tendinopathy, subacromial impingement syndrome, and decreased glenohumeral mobility. These diagnoses are in line with previous studies among persons with PSSP (15). The intention of OMPT was that all affected structures around the shoulder and clinical signs of impairments of UE should be individually treated, without using compensatory movements or losing control of the facilitated muscles. The participants perceived that the training was performed in a close collaboration with the physiotherapist to solve the pain problem during each training session and adjusted the training continuously. Both participants had observed persisting shoulder problems for several months, but experienced that the OMPT successively led to reduced pain and better arm function. Of importance for the participants was that the intervention effect persisted, which was shown both in the clinical assessments and confirmed in the interviews.

Previous studies in patients with shoulder pain due to other causes than PSSP have reported beneficial effects of OMPT. In a review article (52) favourable outcome for supervised strengthening exercises was reported in patients with subacromial impingement and non-specific shoulder pain. Also, guided exercises and joint mobilization (53), and dry needling in combination with eccentric-concentric exercises (54) have been reported as beneficial. In contrast to these specified interventions, common PSSP interventions in neurorehabilitation (50) are often of a more general character (such as positioning, ROM, and strength exercises) and not directed to the specific anatomical structures. However, also specific interventions for PSSP are described in the literature (23), and positive effects are reported for acupuncture, orthosis, and botulinum toxin (25), although some studies were small. But these previous studies had more focus on pain-relieving methods than on identifying and treating the underlying causes of the pain, leading to the possibility that the pain relief might have been temporary.

### Strength and limitations

A strength of our study is that both participants showed substantially reduced PSSP and improved functioning post-intervention, which lasted over the follow-up period. It was observed in the clinical outcome measures, which have shown sound psychometric properties (36, 38, 39, 41, 44) and confirmed in the interviews. The study is limited by the small sample size and no control group; therefore, the results should be interpreted with caution. Even though both participants had chronic PSSP and no spontaneous improvement was expected, it is unknown if the pain would have changed if no intervention had taken place. Further studies are warranted to elucidate if OMPT is appropriate for a broader group, especially persons with severe paresis of the UE. These persons often have other disabilities making it difficult for them to comply with the OMPT assessment and intervention. Thus, before OMPT could be more widely used, larger studies are warranted.

To summarize, the 12-week OMPT program with a thorough examination and intervention aiming at precisely treating and training specific weak, inactive, overactive, or immobile structures around the shoulder might have contributed to the positive outcomes in our study. Decreased shoulder pain, increased ROM, and improvements of arm function in daily activities were found, which lasted over the follow-up period.

### Conclusion

OMPT may be a useful intervention to reduce PSSP in persons with mild to moderate UE paresis. This indicates that physiotherapists treating patients with PSSP would benefit from knowledge of OMPT. However, further larger studies are warranted to confirm the result.
